# Spatial Analysis of Ambient PM_2.5_ Exposure and Bladder Cancer Mortality in Taiwan

**DOI:** 10.3390/ijerph14050508

**Published:** 2017-05-10

**Authors:** Hsin-Ling Yeh, Shang-Wei Hsu, Yu-Chia Chang, Ta-Chien Chan, Hui-Chen Tsou, Yen-Chen Chang, Po-Huang Chiang

**Affiliations:** 1 Institute of Population Health Sciences, National Health Research Institutes, Zhunan 350, Taiwan; hsinlingyeh@nhri.org.tw (H.-L.Y.); candy10819@nhri.org.tw (H.-C.T.); ycchang@nhri.org.tw (Y.-C.C.); 2 Department of Healthcare Administration, Asia University, Taichung 413, Taiwan; victor_h@asia.edu.tw (S.-W.H.); ycchang@asia.edu.tw (Y.-C.C.); 3 Research Center for Humanities and Social Sciences, Academia Sinica, Taipei 115, Taiwan; dachianpig@gmail.com; 4 Department of Public Health, China Medical University, Taichung 400, Taiwan

**Keywords:** fine particles, air pollution, Geographic Information Systems, Bladder Cancer, smoking

## Abstract

Fine particulate matter (PM_2.5_) is an air pollutant that is receiving intense regulatory attention in Taiwan. In previous studies, the effect of air pollution on bladder cancer has been explored. This study was conducted to elucidate the effect of atmospheric PM_2.5_ and other local risk factors on bladder cancer mortality based on available 13-year mortality data. Geographically weighted regression (GWR) was applied to estimate and interpret the spatial variability of the relationships between bladder cancer mortality and ambient PM_2.5_ concentrations, and other variables were covariates used to adjust for the effect of PM_2.5_. After applying a GWR model, the concentration of ambient PM_2.5_ showed a positive correlation with bladder cancer mortality in males in northern Taiwan and females in most of the townships in Taiwan. This is the first time PM_2.5_ has been identified as a risk factor for bladder cancer based on the statistical evidence provided by GWR analysis.

## 1. Introduction

Particulate matter (PM) is microscopic solid or liquid matter suspended in the atmosphere. Subtypes of atmospheric particulate matter include suspended particulate matter (SPM), thoracic and respirable particles [1], inhalable coarse particles, which are particles with a diameter between 2.5 and 10 micrometers (μm), fine particles with a diameter of 2.5 μm or less [2] (PM_2.5_), ultrafine particles (PM_10_), and soot. PM_2.5_ is found in secondary production through photochemical reactions from hydrocarbons and oxides of nitrogen in the air [2] or in the emission of motor vehicles [3]. PM_2.5_ also has been confirmed to be associated with health risks including elevated morbidity [4,5,6,7,8] in human populations around the world. Numerous epidemiological studies have shown associations between PM_2.5_ exposure and respiratory-related mortality, and cardiovascular-related diseases [9,10]. Few studies have explored the effect of air pollution on bladder cancer specifically. A study in Spain pointed out that long-term exposure to polycyclic aromatic hydrocarbons (PAHs) and diesel engine emissions from industries near residential areas was associated with higher risk of bladder cancer [11]. In Taiwan, one matched case-control study found a dose-dependent effect of exposure to NO_2_, SO_2_, and PM_10_ on bladder cancer mortality [12]. In Brazil, a correlation was observed between PM_10_ exposure and bladder cancer incidence [13]. In the United States, air pollution ozone days were positively associated with increased bladder cancer mortality [14]. Increased health risks of bladder cancer from exposure to PM_10_ in air were found in most of these studies. However, no report has yet found an association between exposure to the smaller PM_2.5_ and bladder cancer mortality, although in the field of occupation medicine, an association was found between bladder cancer incidence and PAH exposure [15,16], and the concentration of PAHs in air was found to be correlated to PM_2.5_. Thus, PM_2.5_ might play a role in the etiology of bladder cancer. 

Previous studies indicated that risk factors associated with bladder cancer include cigarette smoking, occupational exposure in the textile industry [17], arsenic in drinking water, environmental pollution, sex, and socioeconomic status [18,19]. The age-adjusted bladder cancer mortality in Taiwan ranged from 2.18 to 2.73 per 100,000 population during 2000–2012, and the age-adjusted bladder cancer incidence ranged from 5.93 to 8.06 per 100,000 population [20]. Both the mortality and incidence of bladder cancer were declining during this 13-year period. However, the geographically specific risks of bladder cancer mortality persisted in some areas such as where Blackfoot disease (BFD) is endemic, and exposure to arsenic in well waters as a specific risk factor is well documented [18,19]. Thus, this study was conducted to elucidate the effect of local risk factors on bladder cancer mortality based on the available 13-year mortality data. To show the spatial heterogeneity risk factors, geographically weighted regression (GWR) was employed in data analysis.

## 2. Materials and Methods 

All the data and variables used were retrieved and derived from government statistical data published on websites, thus informed consent was not needed. All results were presented by sex.

### 2.1. Age-Adjusted Mortality Rates

The age-adjusted bladder cancer mortality rates at the township level were downloaded from the website of the Health Promotion Administration, Ministry of Health and Welfare of Taiwan [20]. The World Health Organization’s world standard population in 2000 was used as the reference population. There are 352 townships in 19 cities or counties, on the main island of Taiwan. In order to focus on the effect of PM_2.5_ and other risk factors rather than arsenic exposure, we excluded four BFD-endemic townships located in southwestern Taiwan. The remaining 348 townships were included for further analysis, on the assumption that the arsenic concentrations in drinking water are similar in these townships. The International Classification of Diseases, Ninth Revision (ICD-9) codes 188 were used for the definitions of bladder cancer. The ring map toolbox of ArcGIS was applied to display the spatio-temporal distribution of bladder cancer mortality rate of 19 cities or counties (including 352 townships) from 2000 to 2012 simultaneously [21].

### 2.2. Smoking Rates

The smoking rates were accessed from “Adult Smoking Behavior Surveillance System” of Taiwan [22]. The average smoking rate (% smokers among total population) was computed for each city or county; the limitations of these data have been discussed elsewhere [23].

### 2.3. PM_2.5_ Concentration

The daily levels of PM_2.5_ at 73 monitoring stations from 2006 to 2011 were acquired from the Environmental Protection Administration (EPA) [23]. The annual average concentration of ambient PM_2.5_ (μg/m^3^) was computed for each station. Then the empirical Bayesian Kriging [24] in ArcGIS (ArcMap, version10.3; ESRI Inc., Redlands, CA, USA) was used to interpolate the annual concentrations throughout Taiwan, and the zonal statistics function was used to calculate the average concentration for each township. Finally, we averaged the annual PM_2.5_ concentrations for each township.

### 2.4. Density of Health Care Facilities

The density of health care facilities of each township was used as the proxy indicator for the spatial disparity of health care resources diversity. The mean number of the National-Health-Insurance-contracted hospitals or clinics during 2008–2012 in each township was retrieved as the numerator of the density of health care facilities, and the denominator was the area (km^2^) of each township. The National Health Insurance of Taiwan is a compulsory universal single-payer social health insurance system, which covers over 99% of the population in Taiwan [25]. 

### 2.5. Area Deprivation Index (ADI)

The area deprivation index (ADI) of this present study was composed of the standardized proportion of elementary occupations (including simple farming, forestry, fishing, and animal husbandry) and the standardized drop-out rate of students aged 15 to 17 from school. The ADI formula has been used in Taiwan as a proxy indicator for socioeconomic status [26]. Data were acquired from the population and housing census data of the year 2010 [27].

### 2.6. Percentage of Aboriginal and Elderly Population

The percentage of aboriginal population at the township level from 2008 to 2011 was directly downloaded from the social-economic database of the Ministry of the Interior, Republic of China (Taiwan) [28]. The percentage of the elderly population (aged 65 or older) at the township level was computed from the household registration data of townships from 2000 to 2012. 

### 2.7. Percentage of Workers in Textile Industry

The percentage of workers in the textile industry at the city or county level in 2006 was taken from the industry, commerce, and service census [27]. 

### 2.8. Statistical Analysis 

To understand the relationship between bladder cancer mortality and possible risk factors including smoking, PM_2.5_, density of health care facilities, area deprivation index, and percentages of aborigines, elderly, and textile workers among the population, GWR was carried out to estimate and interpret the spatial variability of their relationships [23,29,30]. GWR is a powerful method for exploring spatial heterogeneity, through extending traditional regression by allowing local variations in rates of change so that the coefficients in the model are estimated at specific locations [31]. Unlike conventional ordinary least-squares regression analysis (OLS), GWR model is a type of local statistic in which the parameter estimations vary over space, and hence each area has different coefficients. The assumption of spatial non-stationarity is made in GWR, which means the correlations between the independent variable and dependent variables are not the same for every area [30]. First of all, R software 3.2.1 (R Development Core Team, Vienna, Austria) [32] was used to run the OLS to determine the correlations between bladder cancer mortality and the risk factors considered. Then, free software GWR 4.0 (Ritsumeikan University, Kyoto, Japan) [33] was used to run the GWR analysis [34]. The coefficients of each explanatory variable and the R-square in both OLS and GWR are summarized in the results. The residual maps and the spatial distribution of the significant explanatory variables after a GWR analysis will be displayed in the results with ArcGIS (ArcMap, version 10.3; ESRI Inc., Redlands, CA, USA). A Local Moran’s I (Local Indicators of Spatial Association, LISA) cluster map of the residuals was created for identifying the clusters which cannot be explained by the current risk factors [23]. The LISA method shows four types of statistically significant high/low clusters, in which a high value is surrounded primarily by high values called a high-high cluster, and so on. Finally, we find the significant predictors of bladder cancer mortality by using the results of difference of criterion after GWR. A negative value of difference of criterion suggests spatial variability in terms of model selection criteria.

## 3. Results

**Figure 1:**
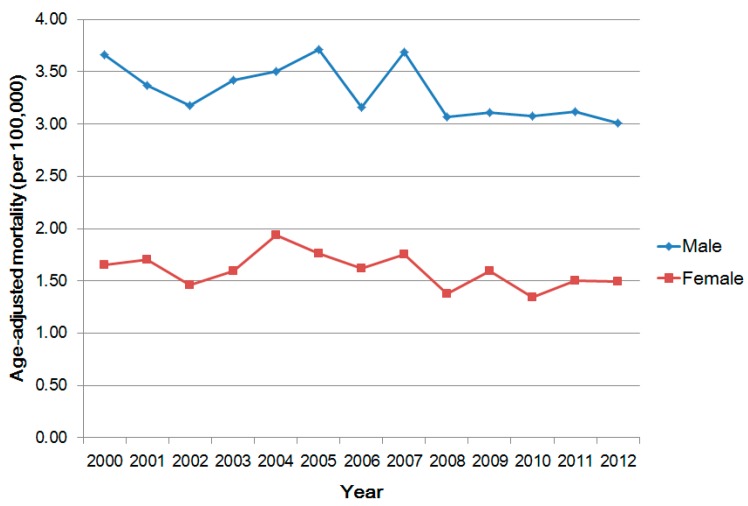
Temporal trend of age-adjusted mortality of bladder cancer in Taiwan, 2000–2012 (Performs chi-squared test for trend in proportions).

**Figure 2:**
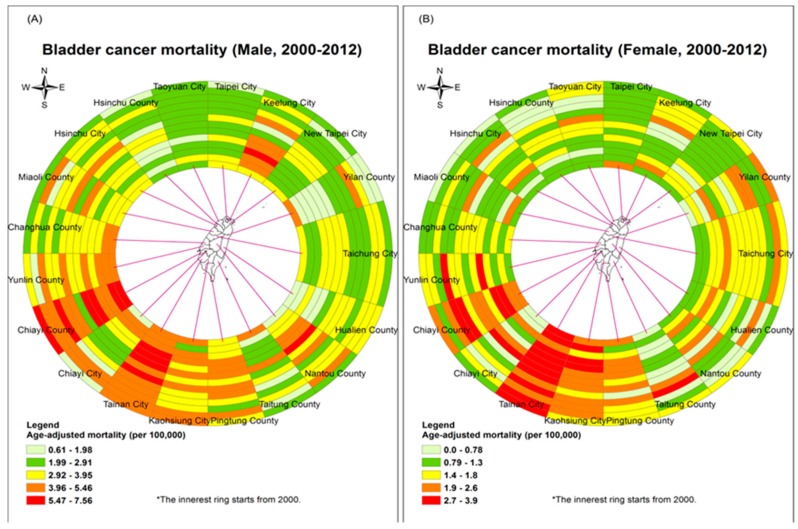
Spatio-temporal distribution of bladder cancer age-adjusted mortality rates with ring map in city or county level during 2000–2012. (**A**) Male (**B**) Female.

The age-adjusted bladder cancer mortality rate in males significantly declined gradually from 3.66 per 100,000 in 2000 to 3.01 per 100,000 in 2012 (*p* < 0.05) and the result for females also significantly declined from 1.65 per 100,000 in 2000 to 1.49 per 100,000 in 2012 (Figure 1, *p* < 0.05). On average, the mortality in males was two times higher than the mortality in females. In Figure 2, the ring maps present the age-adjusted mortality rates of bladder cancer in 19 cities or counties across 13 years. The spatial distribution shows that in southwestern Taiwan, especially Chiayi county and Tainan city, there were persistently higher mortality rates than other cities or counties. The reason is that there were four BFD endemic townships in those two regions, as we mentioned in the method section. Thus, we excluded those four townships to avoid the confounding effect in the following analysis.

In Table 1, the OLS model was applied to demonstrate the relationship between bladder cancer age-adjusted mortality and the risk factors. For males, ambient PM_2.5_ concentration and elderly population showed significant positive correlations with bladder cancer mortality (*p* < 0.05), and the ADI and percentage of the aborigines had significant inverse correlation with bladder cancer mortality (*p* < 0.05). The overall adjusted R-square was 9.2%. For females, smoking rate, ambient PM_2.5_ concentration, and health care facilities density displayed significant positive correlations with bladder cancer mortality (*p* < 0.05), but the percentage of aboriginal population demonstrated significant inverse correlation with bladder cancer mortality (*p* < 0.05). The overall adjusted R-square was 13.2%. The variance inflation factor (VIF) values were all below 7.5, which indicates that there was no collinearity problem.

We used the quartile method to represent the distribution of coefficients across the 348 townships in Table 2. The summary statistics of the coefficients after applying GWR for every township are listed. For the males, the medians of three factors’ coefficients including smoking rate, health care facilities density, and the population of elderly are all positively correlated with bladder cancer mortality. The overall adjusted R-square was 16.2%. For the females, the medians of smoking rate, PM_2.5_, health care facilities density, percentage of working in textile industry, and the percentage of the elderly population were all positively correlated with bladder cancer mortality. The overall adjusted R-square was 20%.

**Figure 3:**
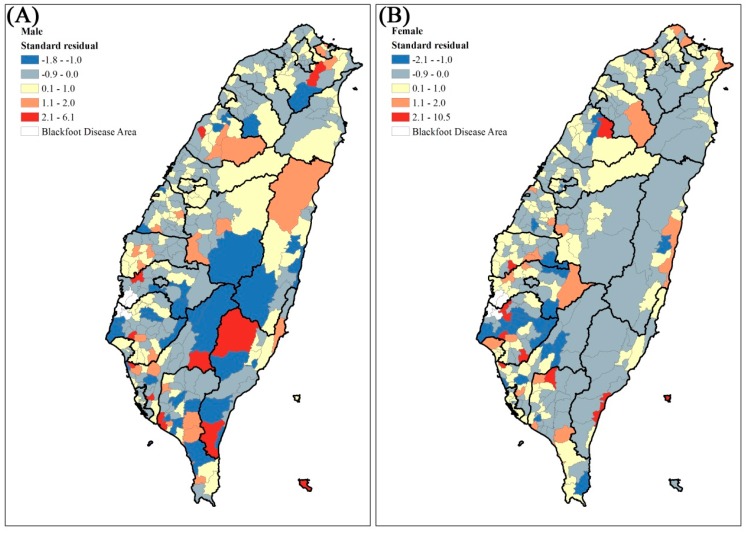
The residual maps for bladder cancer age-adjusted mortality rates by geographically weighted regression from 2000 to 2012. (**A**) Male (**B**) Female.

**Figure 4:**
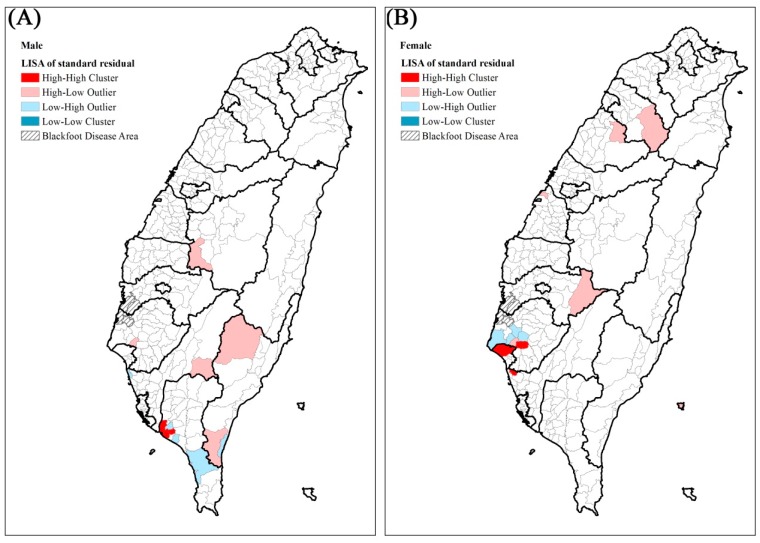
Local Moran’s I of the residuals by geographically weighted regression from 2000 to 2012. (**A**) Male (**B**) Female.

The residual maps after GWR (adjusted for all the above variables) are shown in Figure 3. They indicate that the standard residual in many townships remained larger than 2. Thus, we used local Moran’s I to identify the local high-high clusters of residuals. For both males and females (Figure 4), the high-high clusters were all in southern Taiwan, including Kaohsiung City and Tainan City.

**Figure 5:**
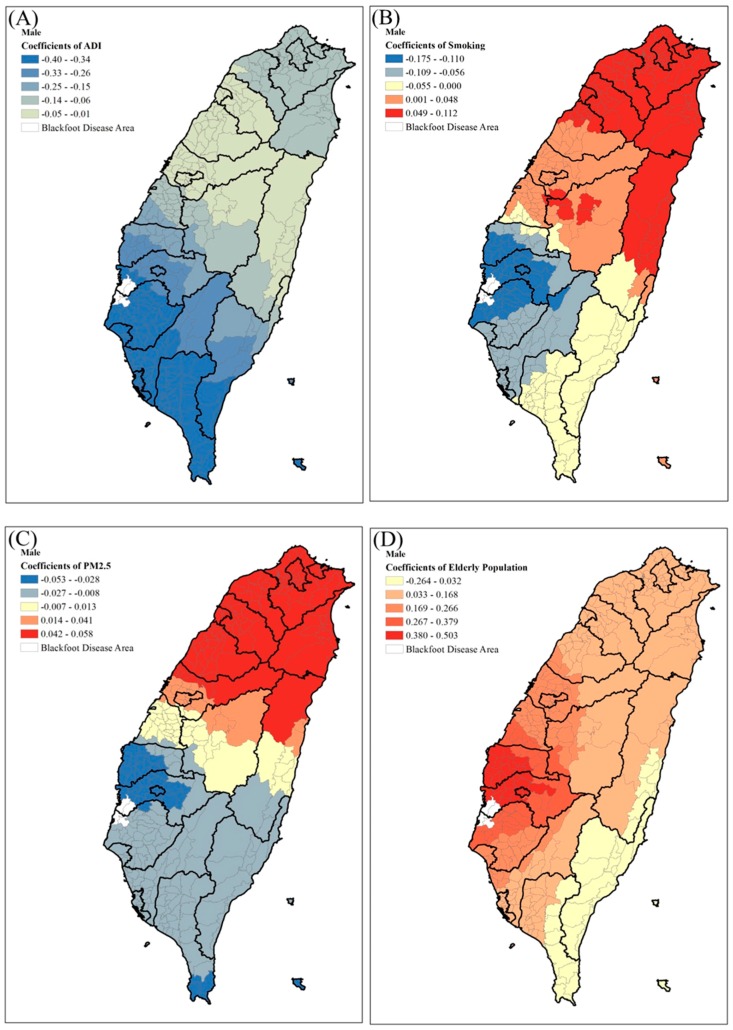
Geographical distributions of the selected significant predictors of male bladder cancer mortality. (**A**) Area Deprivation Index (ADI); (**B**) Smoking rates; (**C**) PM_2.5_ concentration; (**D**) Elderly population.

**Figure 6:**
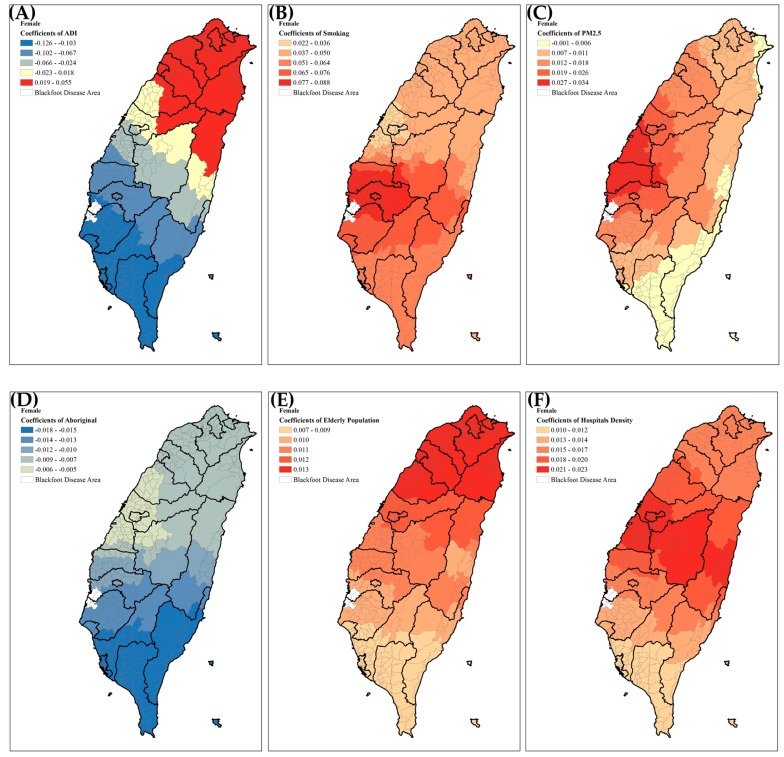
Geographical distributions of the selected significant predictors of female bladder cancer mortality. (**A**) Area Deprivation Index; (**B**) Smoking rates; (**C**) PM_2.5_ concentration; (**D**) Aborigines; (**E**) Health care facilities; (**F**) Elderly population.

In order to show the effects of the significant risk factors for the mortality rates of bladder cancer, we displayed the spatial distribution of four significant predictors of bladder cancer mortality in males (Figure 5). In males, the smoking rate and PM_2.5_ both had higher positive correlations with bladder cancer mortality in northern Taiwan; although the area deprivation index showed negative correlation with bladder cancer mortality, especially in southern Taiwan. However, the population of elderly males showed positive correlation with bladder cancer mortality, especially in central and southern Taiwan. In females (Figure 6), smoking rate, PM_2.5_, health care facilities density, and the population of elderly all showed positive correlations with bladder cancer mortality; area deprivation index showed a positive effect only in northern Taiwan, while the percentage of female aborigines showed a negative effect throughout all of Taiwan.

## 4. Discussion

This paper presents a 13-year spatial-temporal distribution of age-adjusted bladder cancer mortality, and identifies possible cluster areas for future epidemiologic investigation. In this study, we first excluded the arsenic exposure in four BFD townships to disentangle the effects between PM_2.5_ and arsenic exposure on bladder cancer mortality. Spatial variation of the risk factors indeed existed in this study. Thus, the overall R-square performance was elevated 7% from OLS to GWR. Although the temporal trend of bladder cancer mortality was declining, we still found some local clustering townships in southern Taiwan which could not be explained by the current explanatory variables.

In accordance with previous studies, this study has found that elderly male subjects were more prone to develop bladder cancer than females. Aside from age and sex, there are several other risk factors for bladder cancer, including smoking, industrial exposure, certain medicines or herbal dietary supplements, arsenic in drinking water, lack of fluids intake, etc. [35]. Among the risk factors analyzed in this study, the smoking rates of both sexes were positively correlated with bladder cancer mortality. However, the spatial effects of smoking rate were different. In males, the effect in northern Taiwan was higher, but in females, the effect in southern Taiwan was higher. In the results of one meta-analysis [36], cigarette smoking had a higher probability of inducing a more malignant type of urothelial carcinoma and an increased risk of dying due to bladder cancer.

PM_2.5_ had significant positive correlation with bladder cancer mortality in the OLS analysis. From the GWR analysis, PM_2.5_ had higher positive correlation with bladder cancer mortality in males in northern Taiwan and females in central Taiwan. In females, bladder cancer mortality in most of the townships showed a positive correlation with PM_2.5_. In one of the previous studies, bladder cancer mortality in America was found to be higher in areas with high population density, suggesting that a possible cause might be air pollution [37]. Results of one case-control study in Taiwan also showed that NO_2_, SO_2_, and PM_10_ all had significant effects on bladder cancer mortality [12].

The GWR result indicates that health care facility density is positively correlated with bladder cancer mortality in both sexes. This may be attributed to highly urbanized areas with high density of healthcare institutions [38] and air pollution [37].

The ADI of the present study can represent the urbanization level of townships in Taiwan, because it included the standardized proportion of elementary occupations and the standardized school drop-out rate of students aged 15 to 17. The higher ADI is, the less the urbanization of the township, and the fewer pollutants there are from industries and traffic. This might explain why ADI is negatively correlated with bladder cancer mortality in males. Females from less urbanized townships having higher bladder cancer mortality could be attributable to indoor PM_2.5_ exposure. Other than gas and electricity, wood and charcoal were also commonly used decades ago as cooking fuels in rural townships in Taiwan, and charcoal is still popular for barbecues in restaurants and at home on certain holidays. These fuels can produce vast amounts of indoor PM_2.5_ [39]. However, the relationship between ADI and bladder cancer mortality is worth clarifying using a different study design. 

## 5. Limitations

This study demonstrated correlations between some variables and bladder cancer mortality. Like most other studies, we assumed the values at monitoring stations were highly correlated with values at breathing zones [40,41], and all subjects are assumed to have had the same exposures in the same area, estimated by mean concentrations. There are some limitations in this study. First, because of the long latency of bladder cancer and the fact that the bladder cancer mortality data precede the period for which monitored PM_2.5_ data became available, we have to assume that the relative magnitude of exposure concentrations among these townships remained constant over past decades. This assumption seems reasonable because most of the increase in PM_2.5_ is due to the rapid increase in motor vehicles, and the relative rate of increase of motor vehicle ownership among these townships should remain roughly similar over the years. The second limitation is a lack of personal medication information. Traditional Chinese medicines and dietary supplements containing aristolochic acid have been linked with an increased risk of urothelial cancers, including bladder cancer [42,43], and these medicines were not banned in Taiwan until 2013.

Type 2 diabetes is one of the leading causes of death in Taiwan. Oral antidiabetic agents are often prescribed to patients suffering type 2 diabetes to control blood glucose concentration. Results of population-based cohort studies show that pioglitazone, an antidiabetic medication, is associated with an increased risk of bladder cancer [44,45]. In addition, the spatial unit of smoking rates was the city or county level rather than township. Therefore, the heterogeneities within a city or county would be underestimated due to this limitation of the data. However, we tried to evaluate the result by using lung cancer mortality which is based on township to replace smoking rates. Both results were similar (Table A1 and Table A2). For a future study, we plan to use lung cancer data to adjust the smoking rate to the township level. Other lifestyle factors such as intake of fruits and vegetables, taking vitamins and antioxidant supplements, and physical activity, which are factors protecting against bladder cancer, have been reviewed recently [46]. Furthermore, there is a lack of relevant health data, such as disease history and obesity. After controlling for age, sex, and smoking status, subjects with kidney disease had higher bladder cancer mortality [47]. Results of a meta-analysis by Sun et al. [48] showed that individuals with BMI ≥ 30 had higher risk of bladder cancer death. 

## 6. Conclusions 

This study displays the temporal and spatial distribution of bladder cancer mortality in Taiwan. We compared the result of traditional linear model OLS and GWR. The coefficient of determination (adjusted R-square) was higher in the GWR model. Apparently, GWR can explain the correlation between disease mortality and environment risk factors by considering the spatial heterogeneity. After applying a GWR model, the concentration of ambient PM_2.5_ showed positive correlation with bladder cancer mortality in males in northern Taiwan, and females in most of the townships in Taiwan. Having examined the results, there are still some abnormal clusters in southern Taiwan. This will need further research to clarify the relationship between local risk factors and bladder cancer mortality.

## Figures and Tables

**Table 1:** Explanatory factors for bladder cancer age-adjusted mortality rates by ordinary least squares model at township level, Taiwan, 2000–2012. (Blackfoot disease (BFD)-endemic township excluded, *N* = 348).

Variables	Coefficient	S.E.	*p*-Value	VIF
**Male**				
Area deprivation index	−0.181	0.079	0.022 *	1.267
Smoking rates (%)	0.050	0.034	0.146	1.551
PM_2.5_ concentration (μg/m^3^)	0.045	0.012	<0.001 *	1.155
Aborigines (%)	−0.010	0.004	0.020 *	1.307
Health care facilities (per km^2^)	0.003	0.007	0.708	1.227
Workers in textile industry (%)	0.006	0.022	0.805	1.364
Elderly population (%, age ≥ 65)	0.172	0.067	0.011 *	1.289
Adjusted R-square	9.2%			
**Female**				
Area deprivation index	−0.062	0.050	0.216	1.451
Smoking rates (%)	0.064	0.039	0.100	2.294
PM_2.5_ concentration (μg/m^3^)	0.036	0.010	0.001 *	2.042
Aborigines (%)	−0.011	0.003	<0.001 *	1.332
Health care facilities (per km^2^)	0.010	0.004	0.021 *	1.144
Workers in textiles industry (%)	0.008	0.013	0.566	1.234
Elderly population (%, age ≥ 65)	0.025	0.028	0.381	1.480
Adjusted R-square	13.2%			

* *p*-value < 0.05, statistical significance. PM_2.5_, Fine particulate matter; VIF, variance inflation factor.

**Table 2:** The explanatory factors for bladder cancer age-adjusted mortality rates by geographically weighted regression at township level, Taiwan, 2000–2012 (BFD-endemic townships excluded).

Variables	*N*	1st Quartile	Median	3rd Quartile	R. STD.
**Male**					
Area deprivation index	348	−0.363	−0.114	−0.068	0.219
Smoking rates (%)	348	−0.070	0.022	0.061	0.097
PM_2.5_ concentration (μg/m^3^)	348	−0.020	−0.002	0.056	0.056
Aborigines (%)	348	−0.017	−0.016	−0.013	0.003
Health care facilities (per km^2^)	348	−0.005	0.007	0.013	0.013
Workers in textiles industry (%)	348	−0.024	−0.014	0.011	0.026
Elderly population (%, age ≥ 65)	348	0.121	0.143	0.230	0.081
Adjusted R-square	16.2%				
**Female**					
Area deprivation index	348	−0.113	−0.060	0.028	0.105
Smoking rates (%)	348	0.042	0.052	0.065	0.017
PM_2.5_ concentration (μg/m^3^)	348	0.007	0.012	0.022	0.011
Aborigines (%)	348	−0.015	−0.009	−0.008	0.005
Health care facilities (per km^2^)	348	0.013	0.015	0.018	0.004
Workers in textile industry (%)	348	0.005	0.008	0.016	0.008
Elderly population (%, age ≥ 65)	348	0.009	0.011	0.012	0.003
Adjusted R-square	20%				

Note: R. STD. = Robust standard error.

## Appendix A

**Table A1:** The explanatory factors for bladder cancer age-adjusted mortality rates by ordinary least squares model at township level, Taiwan, 2000–2012 (BFD-endemic township excluded, *N* = 348).

Variables	Coefficient	S.E.	*p*-Value	VIF
**Male**				
Area deprivation index	−0.192	0.078	0.014 *	1.267
Lung cancer mortality	0.042	0.012	<0.001 *	1.145
PM_2.5_ concentration (μg/m^3^)	0.048	0.012	<0.001 *	1.163
Aborigines (%)	−0.008	0.004	0.043 *	1.293
Health care facilities (per km^2^)	0.003	0.006	0.067	1.159
Workers in textile industry (%)	−0.029	0.021	0.168	1.212
Elderly population (%, age ≥ 65)	0.185	0.065	0.004 *	1.224
Adjusted R-square	11.7%			
**Female**				
Area deprivation index	−0.049	0.049	0.314	1.442
Lung cancer mortality	0.058	0.013	<0.001 *	1.217
PM_2.5_ concentration (μg/m^3^)	0.026	0.008	<0.001 *	1.194
Aborigines (%)	−0.015	0.003	<0.001 *	1.513
Health care facilities (per km^2^)	0.010	0.004	0.019 *	1.135
Workers in textiles industry (%)	0.000	0.013	0.975	1.158
Elderly population (%, age ≥ 65)	0.016	0.027	0.567	1.446
Adjusted R-square	17%			

* *p*-value < 0.05, statistical significance.

**Table A2:** The explanatory factors for bladder cancer age-adjusted mortality rates by geographically weighted regression at township level, Taiwan, 2000–2012 (BFD-endemic townships excluded).

Variables	*N*	1st Quartile	Median	3rd Quartile	R. STD.
**Male**					
Area deprivation index	348	−0.330	−0.135	−0.058	0.201
Lung cancer mortality	348	0.017	0.045	0.069	0.039
PM_2.5_ concentration (μg/m^3^)	348	−0.016	0.003	0.061	0.057
Aborigines (%)	348	−0.015	−0.012	−0.010	0.004
Health care facilities (per km^2^)	348	−0.006	−0.001	0.016	0.016
Workers in textiles industry (%)	348	−0.038	−0.021	0.046	0.062
Elderly population (%, age ≥ 65)	348	−0.141	0.091	0.213	0.262
Adjusted R-square	21.5%				
**Female**					
Area deprivation index	348	−0.088	−0.044	0.023	0.083
Lung cancer mortality	348	0.049	0.050	0.054	0.003
PM_2.5_ concentration (μg/m^3^)	348	0.002	0.008	0.016	0.010
Aborigines (%)	348	−0.018	−0.012	−0.011	0.005
Health care facilities (per km^2^)	348	0.013	0.015	0.018	0.004
Workers in textile industry (%)	348	−0.002	−0.001	0.003	0.004
Elderly population (%, age ≥ 65)	348	0.009	0.010	0.010	0.001
Adjusted R-square	22.5%				

Note: R. STD. = Robust standard error.
